# Adaptive Strategies for Materials Design using Uncertainties

**DOI:** 10.1038/srep19660

**Published:** 2016-01-21

**Authors:** Prasanna V. Balachandran, Dezhen Xue, James Theiler, John Hogden, Turab Lookman

**Affiliations:** 1 Theoretical Division, Los Alamos National Laboratory, Los Alamos, NM 87545, USA; 2 State Key Laboratory for Mechanical Behavior of Materials, Xi’an Jiaotong University, Xi’an 710049, China; 3 Intelligence and Space Research, Los Alamos National Laboratory, Los Alamos, NM 87545, USA; 4 Computer and Computational Sciences, Los Alamos National Laboratory, Los Alamos, NM 87545, USA

## Abstract

We compare several adaptive design strategies using a data set of 223 M_2_AX family of compounds for which the elastic properties [bulk (B), shear (G), and Young’s (E) modulus] have been computed using density functional theory. The design strategies are decomposed into an iterative loop with two main steps: machine learning is used to train a *regressor* that predicts elastic properties in terms of elementary orbital radii of the individual components of the materials; and a *selector* uses these predictions and their *uncertainties* to choose the next material to investigate. The ultimate goal is to obtain a material with desired elastic properties in as few iterations as possible. We examine how the choice of data set size, regressor and selector impact the design. We find that selectors that use information about the prediction uncertainty outperform those that don’t. Our work is a step in illustrating how adaptive design tools can guide the search for new materials with desired properties.

Regression methods have provided a foundation for modeling structure-property relationships in materials science^1^,^2^,^3^,^4^,^5^,^6^. These methods take as input a data set of known material compositions along with some *property* (e.g. strength, band gap, melting temperature, etc). Each material, in turn, is described in terms of one or more *features* that represent aspects of structure, chemistry, bonding and/or microstructure in an abstract, high-dimensional space. The success of regression is based on its ability to capture the relative variation in a property as a function of the features, eventually culminating in the prediction of new materials with desired properties.

Materials design is an optimization problem with the goal of maximizing (or minimizing) some desired property of a material, denoted by *y*, by varying certain features, denoted by *x*. Optimizing a material for targeted applications generally proceeds by iterating three steps: 1) develop a model (or a set of models) that enables prediction of *y* from *x* of known materials, 2) based on these models, optimally select a value of *x*, which corresponds to a new and yet-to-be synthesized material, and 3) measure *y* at this value of *x*, and add (*x*, *y*) to the database of known properties. The primary bottleneck in material design is step 3, measuring *y*, because it requires synthesis and characterization of new materials, which can be expensive and time consuming. Thus, we want to synthesize as few compounds as possible. Alternatively, in the case of a complex code (e.g. high-throughput first principles calculations^7^,^8^,^9^,^10^), we would want to make as few function calls or tractable number of calculations as possible. Thus, using the experiment or complex code as a means to exhaustively search (in a brute force manner) the vast space to optimize a given property is to be avoided. Instead, it is necessary to use a relatively cheap surrogate or inference model to learn from the available data, and in conjunction with some form of design, guide experiments to minimize the number of new materials that need to be tested. Currently, the work in materials informatics is largely based on performing step 1, which requires finding a *regressor* that fits existing data to predict the best *y*.

Advances in operations research and machine learning suggest that merely choosing the predictions out of the regressor is not the best way to select *x* when the predicted values have uncertainties^11^. This is relevant for materials design because relatively small data sets are often used to extrapolate to a vast unexplored search space^6^, which means that model *uncertainties* will be important. When we can estimate the errors associated with the *y*-values, it is typically better to choose the *x* that gives the best “expected improvement”—a value that depends on both the predicted *y* and the predicted error in our estimate of *y*. The advantages of maximizing the expected improvement are amplified when the function predicting *y* from *x* has local optima—local optima are difficult to escape by many optimization methods. Both Efficient Global Optimization (EGO)^11^,^12^,^13^ and the closely related Knowledge Gradient (KG) algorithms^14^ use information about prediction error to select the next *x* to test. Both EGO and KG show good performance on a variety of problems outside of materials design^11^,^15^, particularly on engineering related problems, and can be used to escape local optima.

In this paper, we describe our studies of the merits of different regressors and selectors for materials design. We briefly discuss various regression approaches in the Section entitled Regression. The next section then focuses on Selectors that use prediction uncertainties to suggest the next material to be tested. We discuss our application of these techniques to representative but well-understood materials science problems in the Section named Elastic Modulus Problems—selecting the compositions of compounds belonging to the family of M_2_AX phases, where the properties of interest are the elastic moduli: bulk (B), shear (G), or Young’s (E) modulus.

## Regression

We studied three regressors: a Gaussian Process Model (GPM)^16^, Support Vector Regression with a radial basis function kernel 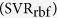 and Support Vector Regression with a linear kernel 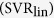. The regressors were implemented in the Scikit-learn python package^17^. Like other types of regression, a GPM assumes that 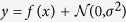 where *f* is the function that must be estimated. Unlike most regression, *f* is treated as a point in an infinite-dimensional Gaussian distribution (that is, a Gaussian *Process*) over the set of functions. The Gaussian Process (like Gaussian Distributions) is completely defined by its mean and covariance, but the mean and covariance are functions and the covariance is given in terms of distances between *x* values. For our studies, the mean function was 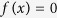 and the covariance was the square exponential function: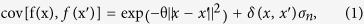 where *θ* and 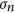 are free parameters. We set *θ* using maximum likelihood. We set 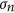 (sometimes called the nugget) using cross-validation. Letting 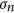 be non-zero can improve generalization when *f* is a rough function. We found that our results improved significantly when 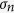 was allowed to be non-zero. With the fully specified Gaussian Process, the distribution of 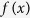 given the training samples is Gaussian with a mean and standard deviation that we can calculate. The calculated mean and standard deviation serve as the predicted *y* value and estimated error around the prediction.

On the other hand, we have observed better performance with the SVR regressors^5^. Here, the regressor is of the form
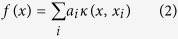
 where the *a*_*i*_’s are coefficients that are fit to the data, and 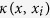 is a kernel function. For 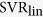, the kernel function is a dot product of *x* and *x*_*i*_, leading to a function 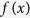 that is linear in *x*. For 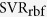, the kernel function is a radial basis function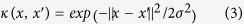 and *σ* is determined by cross-validation. To obtain uncertainties with the SVR models we used a bootstrap approach. Using different subsamples of the training data, we re-trained the regressors multiple times, and maintained an ensemble of these regressors. For a new material, we applied each member of the ensemble, and kept track of the mean and standard deviation of various predictions of each of the predictors in the ensemble. These mean and error estimates were what we fed into the selector algorithms. For all of these learning algorithms, cross-validation was used to optimize the input parameters. Indeed, a separate cross-validation (and distinct parameter estimates) was done for each run.

In addition to the SVR and GPM regressors discussed in this paper, the kernel ridge regression (KRR) method is also used in the materials informatics literature^4^. KRR is similar to SVR_rbf_. Both schemes formulate the solution as a linear combination of Gaussian kernel functions, as expressed in Equation 2, but the coefficients 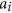 differ. KRR minimizes a quadratic loss function, whereas SVR_rbf_ is based on a (piecewise) linear loss function, potentially making it more robust to outliers. Overall, however, we would not expect substantial differences between them.

## Selectors

In selecting the material to test next, we are implicitly balancing the goal of finding the best material (*exploitation*) with the need to improve our predictive model by *exploration* of the input space. While this balance has been extensively discussed in the literature under *multi-armed bandits* and *surrogate-based optimization*^14^,^15^, for most problems the best solution is not known.

Two heuristic approaches to balancing exploration and exploitation are the selector used by EGO and KG, mentioned in the Introduction. Both EGO and KG assume that we know, for each value of *x*, the probability density function, 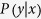, of possible *y* values given that a compound with features *x* is measured. EGO suggests measuring the *x* that maximizes the *expected improvement* in *y* over our current best value. If 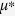 is the value of the best material measured so far, then the expected improvement we would get by testing 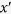 is: 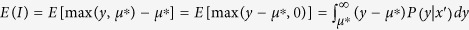. If 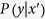 is a Gaussian distribution with mean *μ* and variance 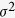, the expected improvement is given by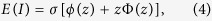 where 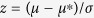 and 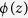 and 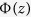 are the standard normal density and cumulative distribution functions, respectively^11^.

**Figure 1:**
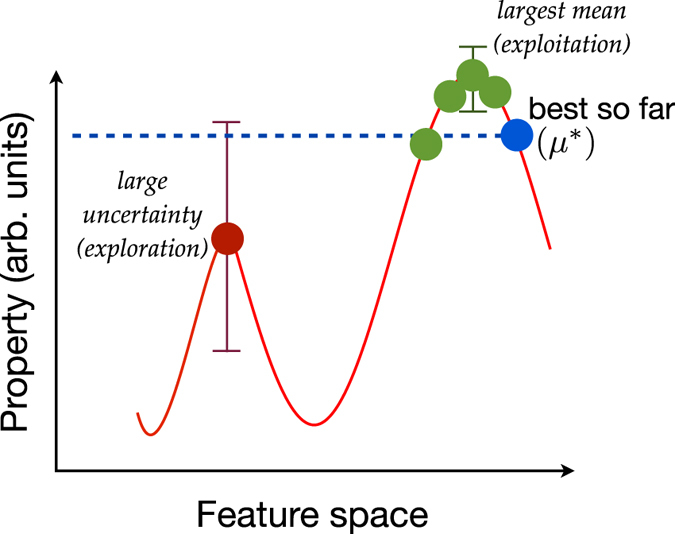
Schematic showing the trade-off between exploration and exploitation. A hypothetical fitness landscape (shown as continuous red line) from the regression model in an abstract high-dimensional feature space with two local maxima. One of the maxima has a data point with a relatively large value for mean, but small uncertainty (error bar). This is due to its close proximity to the best material that is known so far in the training set (blue circle), where the regression algorithm has trained well. The other local maximum, in contrast, has a data point with small mean value (red circle), but relatively large uncertainty. The choice between the selection of data point with the largest mean or largest uncertainty is made by calculating the expected improvement 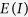, using Equation 4. Consequently, the data point corresponding to the largest 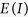 is recommended for the next experimental/theoretical measurement or validation. The key outcome is that 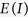 provides a principled basis for optimal learning, because it guides the experimental design in search of materials with desired properties by balancing the trade-off between exploration and exploitation. Horizontal dotted blue line is a guide to the eye showing the data point with the best property measured (or calculated) so far 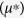 in the training data set.

If all the data samples have the same small uncertainty *σ*, then 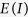 varies monotonically with *μ*, and and maximizing 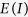 leads to the same selection as simply choosing the maximum prediction. However, increasing *σ* increases 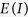 if *μ* is held constant. This follows from Equation (4) where the two limiting conditions that maximize 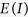 are: (i) small *σ* for a given *μ*, or large *μ* for a given *σ* or (ii) large *σ*, given *μ*. For (i), the expected improvement, 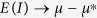. This means that in the limit of no uncertainty or if uncertainties are the same for all points, 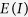 is maximized by choosing the largest *μ* (i.e. exploitation). So the next best material for measurement is chosen by faithfully “trusting” the predictions from the regressor. On the other hand for (ii), 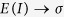 for *σ* large. That is, in the limit of high uncertainty, 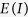 is maximized by choosing the next best material with the largest uncertainty (exploration). It represents a region in our search space, where the regressor is most uncertain. Thus, 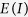 typically captures the relative competition between these two extremes and provides a quantitative estimate to evaluate where the next measurement should be performed. This is shown schematically in Fig. 1.

In EGO, we easily distinguish between measured values (which we assume are precisely known) and unmeasured values that are estimated, with a *μ* and *σ*. In the EGO scenario, there is no need to measure the same material twice. In contrast, KG’s formulation incorporates measurement error. When measurement errors are included, all compounds, measured or not, are on a more equal footing than in EGO—they are all characterized by a distribution of possible values. Having no clear distinction between measured and unmeasured data values, KG compares the mean for each potential compound, 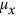, (measured or unmeasured) to the best of the rest of the potential compounds, i.e., 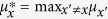. In our work to date, we set the measurement error to 0, so the primary difference between EGO and KG is the difference between 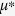 (for EGO’s selector) and 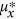 for (KG). In addition to studying the EGO and KG selectors, we studied the selectors defined below:

- Max: This just chooses the highest expected score from the regressor.
- Max-A: Alternates between choosing the material with the highest expected score and the material with the most uncertain estimated score.
- Max-P: Maximizes the probability that a material will be an improvement, without regard to the size of the improvement.
- Random: randomly chooses an unmeasured compound.

## Elastic Modulus Problems

### Data

**Figure 2:**
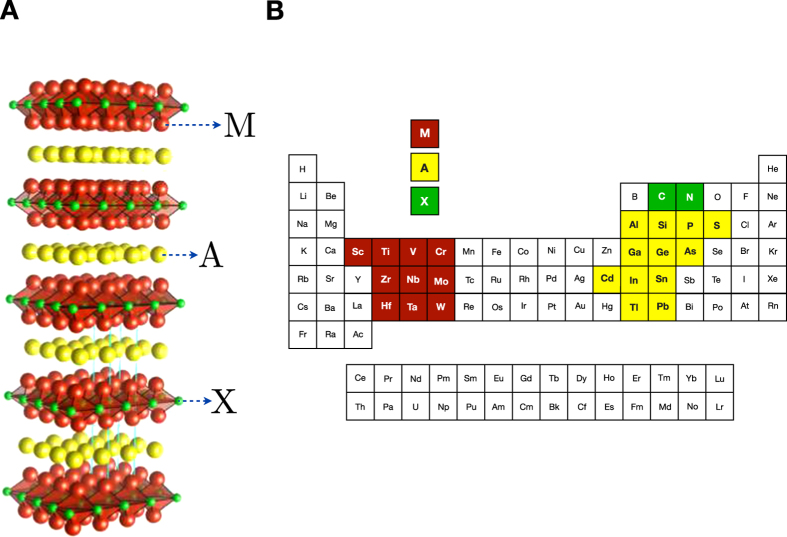
M_2_AX phase for demonstrating the adaptive design strategy. (**A**) Crystal structure of M_2_AX phase in the hexagonal 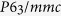 space group. (**B**) The chemical space of M_2_AX phase considered in this work contains about 240 compositions.

Our data set consists of compounds belonging to the family of M_2_AX phases^18^. In the M_2_AX phases, the X atoms reside in the edge-connected M octahedral cages and the A atoms reside in slightly larger right prisms. In Fig. 2, we show the crystal structure and the chemical search space of M_2_AX phases. From Fig. 2B, a total of 240 chemical compositions can be exhaustively enumerated. We separately consider the problems of maximizing the elastic moduli: bulk (B), shear (G), or Young’s (E) modulus. Recently, there has been interest in applying informatics-based methods for M_2_AX phases^19^. However, our objective is different. We focus mainly on the problem of maximizing G, but we will analyze the adaptive design strategies in the context of the other properties as well (not explored by Sitaram *et al*.^19^). The elastic moduli were estimated using density functional theory (DFT) calculations and the data was taken from the literature^20^. Cover *et al*.^20^ carried out DFT as implemented in the Vienna *Ab initio* Simulation Package (VASP) code using the projector-augmented wave (PAW) core potentials. They treated the electron exchange and correlation within the generalized gradient approximation (GGA). Additional details may be found in Cover *et al*.^20^, who compared calculated elastic constants with experimental measurements and report variations up to 30 GPa. The elastic moduli have been calculated for all 240 stoichiometric M_2_AX compounds making this an ideal data set to investigate the nuances of our strategy. Of the 240 M_2_AX compositions, 17 were computed to have negative elastic constants and so were removed from the data set as they violated the Born criteria for elastic stability. This left us with *N* = 223 compounds to study (see Supplemental Information for the dataset).

### Features

We employ orbital radii of M, A, and X-atoms from the Waber-Cromer scale^21^ as features, *x*-values, which include the *s*-, *p*-, and *d*-orbital radii for M, while the *s*- and *p*-orbital radii were used for the A and X atoms. This scale uses the self-consistent Dirac-Slater eigenfunctions and the radii correspond to the principal maxima in the charge-density distribution function for a specific orbital of a neutral atom. These features form a good starting point for this problem because of the fundamental relationship between the electronic charge density and elastic response of materials^22^,^23^. Factors such as elastic anisotropy that classify ductile from brittle materials have been shown to be related to the directional nature (or the lack thereof) of the chemical bonds formed between the *s*-, *p*-, *d*- or *f*-orbitals (near the Fermi level) of the nearest-neighbor atoms^24^. Furthermore, it was recently shown that these features have many characteristics that are favorable for machine learning studies^25^.

## Results

**Figure 3:**
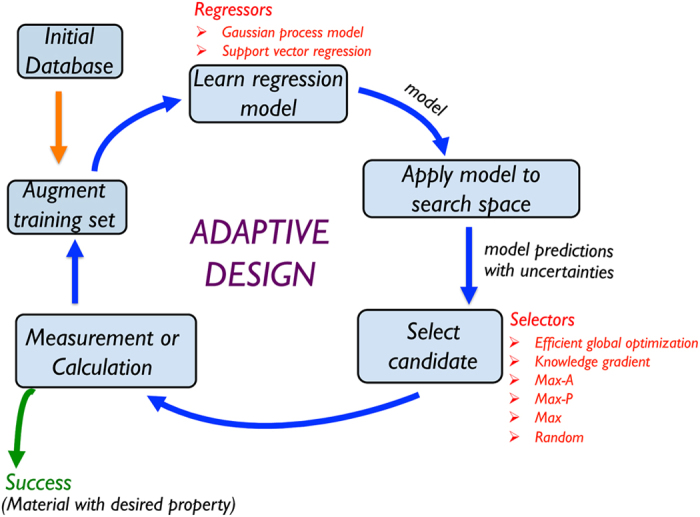
Adaptive design strategy implemented in this work. We start with an initial database of the elastic moduli of M_2_AX compounds calculated from density functional theory (DFT)^20^. From this database, we randomly select a subset of M_2_AX compounds (of varied size) and use it to train a regression model. The rest of the data are hidden from the regressor and we refer to it as “unexplored search space”. We used a Gaussian process model (GPM), Support vector regression with a radial basis function kernel (SVR_rbf_) and Support vector regression with a linear kernel (SVR_lin_) as regressors. The learned regression model is applied to the search space and used to predict the property with uncertainties for the unexplored materials, and a “selector” is used to select a candidate M_2_AX compound by balancing the trade-off between exploration and exploitation. The selectors include KG, Random and Max, Max-A, Max-P, which are variations on EGO (see main text for additional details). The property of the selected candidate is calculated or measured, which in this work is already known. The material is augmented to the training set and a new model is learned. This process typically iterates either a fixed number of steps (*i.e.*, a fixed number of new measurements) or until the material with the desired property is found (Success). Our loop essentially performs a global optimization.

In practice, as seen schematically in Fig. 3, we find a new material by first fitting a regressor to the materials whose properties we already know, and then using that regressor to predict the properties of the materials in our domain of interest. Based on those predictions, a selector picks the most promising candidate. The properties of this candidate are measured (in this case, a DFT calculation is involved; in other cases, this might require synthesizing the material and performing laboratory measurements on that material). If the properties of this material are sufficiently favorable, or if the budget for testing new materials has been depleted, the iteration stops. The result of these iterations is a set of materials with (now) known properties, from which the best can be chosen for the purpose at hand.

In this work we re-run our procedure with statistics maintained for how well on average different regressor/selector pairs perform. This performance is measured both in terms of an “opportunity cost” (defined as the modulus difference between the current-best and the overall-best), and the number of iterations required to “discover” the best material. Iterations-until-best is a good measure of performance because each iteration is expensive and our aim is to minimize the cost. Of course, in practice we would not know what the best value in the overall population or full data set is, so strictly speaking, neither iterations-until-best nor opportunity cost can actually be obtained in an operational scenario. What can be obtained is best-so-far, which is (apart from an offset) equivalent to opportunity cost. Another advantage of opportunity cost is that it is commonly used in the literature (e.g. Powell and Ryzhov^26^ use opportunity cost to compare performance of KG and EGO algorithms). On the other hand, iterations-until-best provides a different perspective on performance; lower opportunity cost generally corresponds to fewer iterations-until-best, but iteration count emphasizes the performance after many iterations.

Each trial starts with a set of *M* initial compounds, chosen at random from 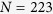 samples in the full data set. The regressor is used to predict a distribution (mean and variance) of values for the property of interest for each of the data samples not in the training set. The *selector* uses these 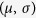 values to pick a promising candidate. This candidate is then measured to determine its actual *y* value. For this study, we know all the *y* values, so this measurement consists simply in revealing the property for that material. The material is added to the training data, and a new regression model is learned from the augmented training data. This process iterates until the material with the “desired” property value is found. We consider several regressor/selector pairs, for a range of initial *M* values, and over 1000 random initial training sets. It is applied to each of the six optimization problems (maximizing and minimizing the three elastic moduli).

**Figure 4:**
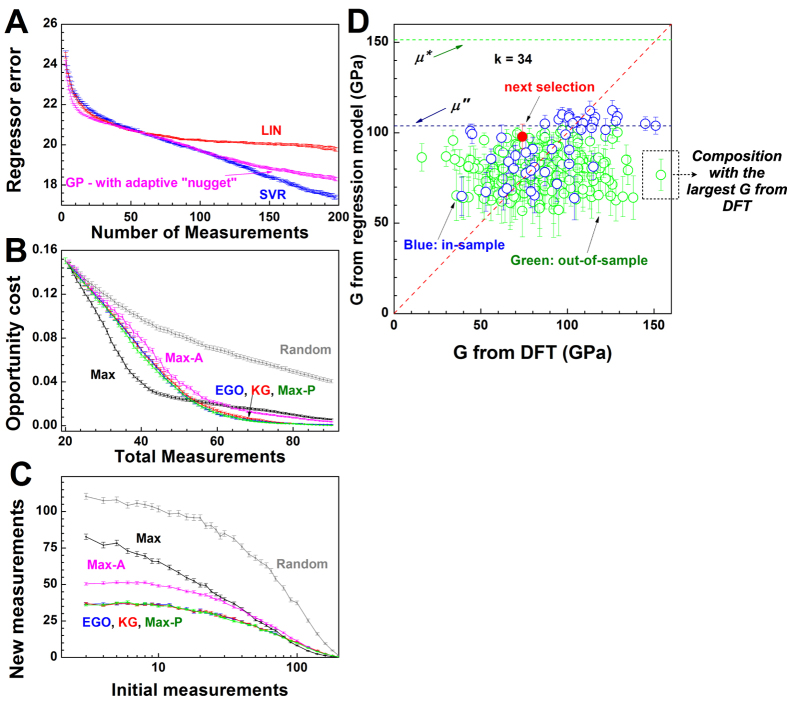
Controlled computational experiments to benchmark and test our design loop. (**A**) Relative performance of three regressors,where the regressor error is the error from cross-validation. (**B**) Normalized opportunity cost for SVR_rbf_ as a function of various selectors. The normalized opportunity cost is the ratio of the difference between the overall best value in the data set and the best-so-far to the range [maximum(G)–minimum(G)] of the shear modulus, G. (**C**) Number of new measurements required to identify the best material as a function of number of initial data points. Results plotted in (**A**–**C**) correspond to mean and standard error (of the mean) over 1,000 trials. (**D**) In a single trial, after twenty initial and fourteen subsequent measurements, an SVR_rbf_ model is fit to the 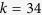 training points (in blue), and applied both to in-sample and out-of-sample data. Here, 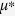 corresponds to the largest DFT value seen so far, and 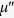 is the largest estimated value. EGO and KG are based on comparisons of predicted values to 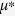 and to 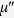. Composition with the largest G value from DFT is highlighted to show that we are in the regime of high model error.

In Fig. 4A, we compare the relative performance of the three regressors by plotting the regressor error as a function of the size of the training set *M*. Error bars represent the standard deviation of the mean for the regressor errors. We find that SVR_rbf_ outperforms GPM (only marginally) and SVR_lin_ (more clearly), which leads to the choice of SVR_rbf_ as the regressor for predicting G. Using this regressor, we then compared selectors. For each selector, we performed 1,000 trials, each initialized with a different randomly chosen set of *M* = 20 compositions. For each trial, the *M* samples were used to train an ensemble of 10 SVR_rbf_ regressors, each regressor being trained from random-with-replacement resampling of the dataset. This bootstrap training enabled the regressor to provide a prediction 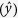 for G along with an error estimate (*σ*), for each composition in the virtual set of candidate samples.

The predicted 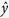 and *σ* then serve as input to the selector, which computes a measure (e.g. expected improvement for EGO) for each candidate material (see Equation 4). The M_2_AX composition with the highest measure is selected as the candidate for the next experiment. The DFT-computed value of G for that candidate is used to augment the training set, and the regression/selection process is iterated for a fixed number of iterations, with each iteration corresponding to a cycle in the loop. A normalized opportunity cost is computed as a function of total measurements in Fig. 4B. Initially, the SVR_rbf_:Max combination outperforms all other SVR_rbf_:selector combinations. After about 50 iterations, however, SVR_rbf_:Max is outperformed by SVR_rbf_:EGO, KG and Max-P, which behave very similarly.

We interpret the different behavior between SVR_rbf_:Max and SVR_rbf_:EGO, KG or Max-P selectors by conjecturing that our SVR_rbf_ regressor is a continuous, non-linear function in the high-dimensional feature space with several local maxima (Fig. 1 shows a schematic of a landscape with two local maxima). During the initial iterations, where there are fewer data points to train SVR_rbf_, the regressor model leads to a local maximum. Even after a relatively large number of iterations (~50), we find that the opportunity cost for SVR_rbf_:Max does not reach zero, suggesting that SVR_rbf_:Max continuously selects candidate M_2_AX compositions that are in the vicinity of the local maximum. Since the Max selector does not take into account uncertainties in the predicted values, it is not able to *explore* the feature search space. Hence, it cannot overcome the local maximum, until a relatively large number of data points that span the chemical space become available to train the regressor. Therefore, SVR_rbf_:Max is not able to rapidly find the optimal M_2_AX composition with the largest G. In contrast, the SVR_rbf_:EGO, KG or Max-P combinations make use of uncertainties. These selectors employ expected improvement (Equation 4), which samples the composition space where the uncertainties are large. As a result, there is a balance between the local and global search, which is reflected in Fig. 4B where after ~50 iterations the opportunity cost for SVR_rbf_:EGO, KG or Max-P is lower than that for SVR_rbf_:Max. Figure 4C shows that when the initial number of random measurements is small, SVR_rbf_:EGO, KG and Max-P all discover the optimal composition with fewer new measurements (~35) than the other selectors (Max, Max-A). Merely choosing measurements at Random does not perform well.

Figure 4D shows a single step in a single trial. The trial started with 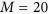 measured values, but after fourteen measurements, there are now 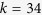 materials with known G modulus. These are used to train the regressor; 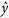 and *σ* are plotted for all of the materials, including those in blue whose true *y* values are known. The next selection is the most promising value based on its 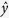 and *σ*. This figure shows that we are in a regime of very high model error. Notice the out-of-sample data points (green in Fig. 4D) and in the limit of an ideal regression model, these points should lie close to the dotted red line. We highlight an out-of-sample data point, whose G value from DFT is 152 GPa, whereas the predicted G (mean value) from the SVR_rbf_ regressor is 76 GPa. Although we would prefer regressor models with lower error, we remark that high model error is likely to be a common situation in data-driven approach to materials design and discovery with small data sets and a vast unexplored search space. We also see that even in the regime of large regressor error, directed search strategies can find optimal materials with fewer iterations than would be found with random selection, and exploitation/exploration trade-off approaches (such as EGO and KG) are more effective than the naive Max selector.

**Figure 5:**
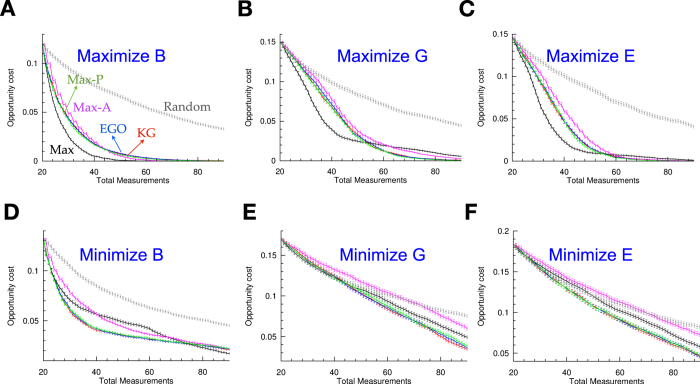
Results from SVR_rbf_: Selector combination which maximize or minimize various elastic properties. (**A**) Maximize bulk modulus, (**B**) Maximize shear modulus (discussed in the article) (**C**) Maximize Young’s modulus, (**D**) Minimize bulk modulus, (**E**) Minimize shear modulus, and (**F**) Minimize Young’s modulus. We utilized the following selectors: Random (grey), Max (black), Max-A (magenta), Max-P (green), Knowledge Gradient (KG) (red) and Efficient Global Optimization (EGO) (blue). In these simulated experiments, we started with an initial number of 20 data points as training set for the regressor. We plot normalized opportunity cost vs. total new measurements required to find the best material with optimal property. Best material with optimal property occurs when the opportunity cost is zero.

To further test the robustness of our iterative design loop and regressor:selector combinations, we performed five additional simulated experiments, for a total of six that involved maximizing and minimizing all the three elastic properties (B, G, and E). In Fig. 5, we show the SVR_rbf_:selector combination results for all elastic properties as a function of the normalized opportunity cost. We selected six different selectors: Random, Max, Max-A, Max-P, Knowledge Gradient (KG) and Efficient Global Optimization (EGO). In the majority of the cases, we found negligible difference between KG and EGO. In all but one case (the exception was maximizing the B modulus, Fig. 5A), KG and EGO outperformed the simple-minded Max selector.

## Discussion

With growing interest in accelerating new materials discovery, machine learning methods are finding increasing use in materials science problems^27^,^28^,^29^,^30^,^31^,^32^,^33^. One of the outstanding challenges involved in a purely data-driven approach that utilizes experimental data is that regression models are trained on a relatively small data set and applied to predict the properties of a vast array of materials in the unexplored chemical space. Ideally, we would prefer highly accurate models, and if such accuracy is attainable, then the model can accurately predict which material has the best property. Our work has shown that such a naïve exploitation strategy often results in suboptimal outcomes. This suboptimality has also been reported in the drug discovery and QSAR literature, and attributed to “activity cliffs” and multiple local optima in the regression fitness landscape^34^,^35^.

We have demonstrated a more robust approach to finding new materials within a global optimization framework using regression with uncertainties, design of experiments (selectors) and iterative feedback, which we collectively refer to as adaptive design. The approach balances the trade-off between exploration and exploitation by choosing potential candidates that maximize the “expected improvement” (or minimize the expected opportunity cost) over the search space. We applied this design strategy on a M_2_AX phase data set obtained from DFT calculations. A smaller regressor error is always preferred, but in data-driven approaches to materials design and discovery, the regressor quality may be limited. In this regime of large regressor error it is important to take that error into consideration in the selection of the next material to investigate. In the data sets that we explored in this paper, the regressor error was substantial, and selector strategies that incorporated exploration (notably, EGO and KG) outperformed the purely exploitive strategy of selecting the material with the best predicted value. We conclude that incorporating exploration into the selection of new materials yields better long-term performance in the context of optimizing targeted properties of new materials.

Finally, we discuss a number of challenges and generalizations for materials design. Although we have demonstrated the nuances and efficacy of the adaptive design approach using a data set from density-functional theory calculations, real materials design problems that involve experimental measurements rarely offer the luxury of precise and error-free property (*y*) values. Furthermore, as the chemical complexity increases (e.g. multicomponent systems), the number of potential crystal structure-types and chemical compositions to explore also increases exponentially. Often, only a small fraction from this vast structural and chemical space is experimentally known. As a result, there are large uncertainties in the unexplored space. Under such circumstances, adaptive design strategies that utilize a selector to suggest materials, which do not necessarily obey the trends of samples in the training data set, could prove to be a vital element for designing new materials. Our work also suggests that the adaptive design is quite forgiving of the quality of the regressor. In addition, the work can be generalized to the case where there are multiple objectives in the design. This is where more than one materials property needs to be optimized simultaneously, for example, an alloy with a small hysteresis to reduce fatigue but a large enough working temperature to be useful. In addition to the model uncertainties that originate from sampling the data and regressors, measurement errors (e.g., from processing conditions, inherent instrumentation limitations) need to be taken into account. The KG design selector, in its general form, can handle measurement uncertainties and can be applied here. Typical experimental materials problems have relatively small data sets and therefore the choice of regressor:selector combinations, as a function of the training data size, needs to be carefully evaluated as it is difficult to assess this *a priori*. It may be that some techniques will work better on certain data sets and for materials design problems with small training data sizes and vast unexplored search spaces, the regressor:selector combinations may need to be reexamined and retrained with every new data point for robustness.

## Additional Information

**How to cite this article**: Balachandran, P. V. *et al*. Adaptive Strategies for Materials Design using Uncertainties. *Sci. Rep.* **6**, 19660; doi: 10.1038/srep19660 (2016).

## Supplementary Material

Supplementary Information
